# Staff experiences of implementing Dementia Care Mapping to improve the quality of dementia care in care homes: a qualitative process evaluation

**DOI:** 10.1186/s12913-021-06152-6

**Published:** 2021-02-12

**Authors:** Alys Wyn Griffiths, Olivia C. Robinson, Emily Shoesmith, Rachael Kelley, Claire A. Surr

**Affiliations:** Centre for Dementia Research, Leeds Beckett University, City Campus, Leeds, LS1 3HE UK

**Keywords:** Psychosocial interventions, Long-term care, Practice development, Staff development, Sustainability, Nursing homes, Residential care, Implementation

## Abstract

**Background:**

Dementia Care Mapping™ (DCM) is a widely used, staff-led, psychosocial intervention to support the implementation of person-centred care. Efficacy evaluations in care homes have produced mixed outcomes, with implementation problems identified. Understanding the experiences of staff trained to lead DCM implementation is crucial to understanding implementation challenges, yet this has rarely been formally explored. This study aimed to examine the experiences of care home staff trained to lead DCM implementation, within a large cluster randomised controlled trial.

**Methods:**

Process evaluation including, semi-structured interviews with 27 trained mappers from 16 intervention allocated care homes. Data were analysed using template variant of thematic analysis.

**Results:**

Three main themes were identified 1) Preparedness to lead - While mappers overwhelmingly enjoyed DCM training, many did not have the personal attributes required to lead practice change and felt DCM training did not adequately equip them to implement it in practice. For many their expectations of the mapper role at recruitment contrasted with the reality once they began to attempt implementation; 2) Transferring knowledge into practice – Due to the complex nature of DCM, developing mastery required regular practice of DCM skills, which was difficult to achieve within available time and resources. Gaining engagement of and transferring learning to the wider staff team was challenging, with benefits of DCM largely limited to the mappers themselves, rather than realised at a care home level; and 3) Sustaining DCM - This required a perception of DCM as beneficial, allocation of adequate resources and support for the process which was often not able to be provided, for the mapper role to fit with the staff member’s usual duties and for DCM to fit with the home’s ethos and future plans for care.

**Conclusions:**

Many care homes may not have staff with the requisite skills to lead practice change using DCM, or the requisite staffing, resources or leadership support required for sustainable implementation. Adaptations to the DCM tool, process and training may be required to reduce its complexity and burden and increase chances of implementation success. Alternatively, models of implementation not reliant on care home staff may be required.

## Background

Within England alone there are around 16,000 registered care homes (nursing and residential) [1], and up to 80% of residents are thought to have dementia [2]. The symptoms of dementia, as well as behavioural reactions to care that fails to meet peoples’ often complex needs, can lead to the occurrence of agitation, aggression, hallucinations, depression, anxiety, and other behaviours that can be difficult for staff to understand and support [3]. Such behaviours can be distressing for the individuals’ experiencing them and can impact negatively on their and other residents’ well-being [4] and on staff. Person-centred care is considered best practice in informing care approaches to prevent, reduce and support ways of working with people with dementia experiencing such behaviours [5] and may also improve working conditions for staff [6]. Person-centred care means providing a supportive social environment where people with dementia are valued, treated as individuals, and staff are encouraged to see the world from the person’s perspective [7].

Within dementia care, psychosocial interventions are increasingly being used to support delivery of person-centred care as an alternative to pharmacological responses that lack efficacy and present significant health risks [8]. Many of these are complex interventions, designed to help identify and provide tailored responses to the underlying needs or cause of a person’s behaviour [9]. For successful implementation, such interventions must be easily integrated into everyday practice, have benefits which are clearly recognisable to staff implementing them, provide opportunities for staff to practice implementation and include adequate on-the-ground support for implementation e.g. through the use of champions or intervention leads [10].

One intervention that includes many of these features is Dementia Care Mapping™ (DCM) [11, 12]. DCM has been used in dementia-care facilities over the last 20 years to help improve the delivery of person-centred care for people living with dementia [13]. It uses structured observations of resident behaviours and well-being, alongside assessment of the quality of staff interactions and care practices, as the basis for evidence-based action planning to support person-centred practice development. It is conducted through applying cycles of preparation and team briefings, observation, data analysis, report writing and feedback to the care team and collective action planning [14]. In the standard model of application those responsible for implementing the intervention (‘mappers’) are trained staff members working in the care setting. They undergo intensive 4-day standardised training in the method as preparation for this role. A staff-led model has the benefit of allowing care homes to implement DCM flexibly and sustainably and for staff who have expertise in DCM to be embedded within a care team, to support implementation and monitoring of action plans. DCM is a ‘whole home’ intervention and so learning from observing a small sample of residents should transfer to the care of all residents within the home [14, 15]. Any care practices observed on the day of mapping observations, whether these be good or poor, are assumed to reflect general practices and are discussed as such during feedback sessions. No staff members on shift during mapping observations are singled out in feedback (except if abusive practice is observed, in which case this would be addressed via usual procedures), in order that staff see the process as developmental for all and the culture of care in a setting, in which poor practices develop is addressed as a whole.

Whilst some studies have reported benefits of use of DCM in care home settings, including reduced agitation, falls and neuropsychiatric symptoms [16, 17] others have found no benefit of DCM over usual care for resident agitation, behaviours that staff may find challenging to support, or quality of life [18–20]. The studies reporting positive outcomes used researcher-led DCM, while those that did not used real-world staff-led cycles, with the authors suggesting that poor staff-led implementation may contribute to a lack of efficacy.

Despite DCM’s widespread and long-term use in care home settings, there remains limited formal exploration of the process of its implementation in practice [21]. Only two previous controlled studies of DCM, have included a process evaluation [20, 22], to qualitatively explore implementation and fidelity. Both used the staff-led model of DCM implementation and found implementation issues within some care home sites. These process evaluations identified the importance of the organizational context and commitment to DCM in supporting implementation, including support from ‘champions’ at team and management levels, strong leadership and co-ordination for DCM activities and ongoing training and support for those trained to use and lead DCM (mappers), as key components for successful implementation. Neither specifically examined the experiences of the mappers leading DCM. Given care home staff trained as mappers have a key role to play in DCM implementation, greater understanding of their experiences and their support needs to successfully implement a complex intervention such as DCM is vital if potential implementation issues are to be identified and addressed. Such understanding is also likely to have relevance for the implementation of other staff-led interventions within care home settings.

## Methods

### Aim

The aim of this study was to examine the experiences of implementing DCM from the perspective of care home staff, trained as mappers, within a large randomised controlled trial.

### Design

This study was part of a process evaluation embedded within the DCM EPIC cluster randomised controlled trial [23] (Trial registration number ISRCTN82288852)**.** The main trial results are published elsewhere [19] as is a discussion of intervention fidelity [24] and the broad barriers and facilitators to DCM implementation experienced by participating care homes (such as managerial turnover, staffing issues and competing priorities) [25]. In summary, the trial involved 50 residential (who provide 24-h care without on-site nurses), nursing (who provide 24- h nursing care) and dementia specialist care homes (who provide either residential or nursing care with staff with specific expertise in dementia) recruited from three areas of England (West Yorkshire, South London, Oxfordshire), with 31 randomised to the DCM intervention and 19 to usual care control.

### DCM implementation

Two staff members from each intervention care home received standardised 4-day training in DCM by attending a course in either Yorkshire or London, which required staying overnight at the training venue or daily travel to attend. The cost of the training, travel, accommodation and meals were paid for by the trial. The mapper selection process consisted of identification of individuals with suitable skills and qualities via researcher conversation with the care home manager, using a set of criteria developed by the research team, based on available guidance on required mapper skills and qualities [12, 14, 15]. These included skills and qualities such as having English language skills commensurate with attending training and writing DCM reports, having suitable IT skills to prepare a basic report and having the confidence to lead meetings to brief and feedback findings to colleagues. These individuals were then approached by the researcher and manager to establish willingness to act as a mapper. Consenting mappers were asked to complete a course booking form that included the dates and venue of the training and a 500-word summary of their understanding and expectations of the role and why they wished to undertake it, to support the trial team in ensuring that all mappers understood and were prepared to undertake the role. This summary was exclusive to the trial and is not usual practice in applications to attend DCM training. Mappers were asked to implement three DCM cycles over fifteen months (3-months, 8-months and 13-months post randomisation). Each cycle involved holding at least one briefing session (for staff, and where possible/appropriate relatives and residents); observing up to five residents with dementia for up to six-hours (numbers observed and period of time for was dependent on their confidence); analysing the data and producing a feedback report in a standardised format; delivering at least one formal feedback session; and producing action plans for each observed resident and one for the whole home/unit. To support standardised intervention implementation an external DCM expert provided practical in-home and remote support throughout the first cycle. The experiences and role of the external expert is explored separately [26]. Overall intervention fidelity was poor. Most care homes (52%) completed only one of the three required DCM cycles, with only 13% of care homes completing the per protocol three cycles to an acceptable level and 10% of homes failing to undertake any DCM activity [24].

### Sample

Purposive sampling was used to select a sub-set of 18 intervention homes which had achieved varying degrees of DCM implementation (0, 1, 2 or 3 cycles) during the trial. These were selected by the trial management team based on return of DCM fidelity documentation during the trial (0 cycles *n* = 3, 1 cycle *n* = 9, 2 cycles n = 3, 3 cycles n = 3). A total of 27 mappers still employed by these care homes at the time of data collection were eligible to participate (7 having left the home during intervention implementation and 2 being unavailable for other reasons). Of these, all 27 agreed to participate (two of whom were also care home managers), representing 16 of the 18 homes taking part in the process evaluation. One home that had completed no cycles and one that had completed 2 cycles were not represented as they had no trained mappers remaining in the home.

### Data collection

**Fig. 1:**
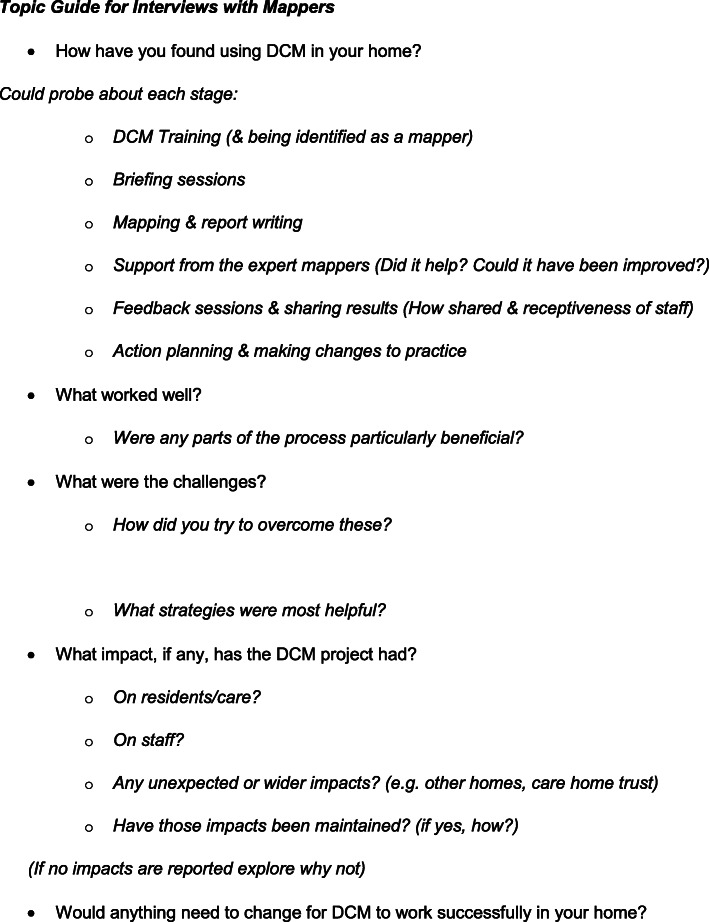
Mapper interview topic guide

Following completion of trial data collection, the process evaluation interviews were conducted using a topic guide developed by the research team (see Fig. 1). The guide was able to be used flexibly by the researchers including use of prompts to request elaboration on important issues. All participants were interviewed either alone or as a pair with their co-mapper, in the care home they worked in, in a quiet place without any residents or other staff members present. Interviews were recorded and transcribed verbatim. The interviews were conducted by a trained researcher based at one the three recruitment hubs.

### Data analysis

Interview data were analysed using template analysis [27]; a form of thematic analysis that uses both top down and bottom up coding. Analysis consisted of an initial set of a priori ‘categories of interest’ that included perceptions of DCM, experiences of and the barriers and facilitators to implementation, as well as open ‘bottom-up’ coding. Two researchers independently coded each transcript line by line. They coded sections of transcript to each of the categories of interest as appropriate and under each category identified themes within the data. After coding an initial set of 11 transcripts the research team discussed and agreed the initial themes and categories and developed a coding template. Two researchers from the team coded each remaining transcript, agreeing and developing the coding within the template through team discussion. Development of the coding template continued throughout data analysis, informed by the developing themes and ongoing discussion.

### Ethical issues

Ethical approval for the study was granted by NRES Committee Yorkshire & the Humber - Bradford Leeds REC ref. 13/YH/0016. All participants gave written, informed consent to participate.

## Results

The majority of mappers were female (85.2%) and most worked in senior roles (70.4%) (e.g. manager, deputy, nurse, team leader, senior care assistant) (see Table 1 for participant demographics), due to the requirement within the trial for mappers to already have a range of skills and qualities to lead DCM implementation. Of the interviews conducted, 23 were individual and two were conducted with a pair of mappers. They ranged from 4 min 58 s (in homes where mappers had been unable to attend DCM training or undertake any DCM activity) to 38 min and 4 s duration (average 14 min 17 s).

**Table 1:** Participant demographics

Demographics (***n*** = 27)	N (%)
Job role	
Manager/deputy manager	7 (25.9)
Team leader/senior careworker	9 (33.3)
Nurse	3 (11.1)
Careworker/activity organiser/cook	8 (29.6)
Sex	
Female	23 (85.2)

The results are presented as three themes ‘*Preparation to lead DCM’, ‘Transferring knowledge into practice’* and ‘*Sustaining DCM’*, each of which have several sub-themes. Illustrative quotes include the care home and participant number and the number of DCM cycles completed.

### Preparedness to lead DCM

Mappers discussed their preparedness to lead DCM under two sub-themes: learning the required skills and the expectations versus reality of the role.

#### Learning the required skills

Generally, mappers reported positive experiences of their DCM training, including, for example, improved knowledge about person-centred care changing their perceptions of people with dementia and dementia care. Most participants stated they enjoyed the interactive training approach and enthusiasm of the trainers for DCM. Mappers in particular noted perceived personal benefits to them of attending training including changing their perspectives on provision of dementia care.

*“The training made you look at things differently within the home. You know we both said the same thing, it does make you look differently.” (10666/10300 0 cycles)*

*“Something about the way that it’s presented, it did, I think for me, fundamentally shift the way I look at dementia.” (50018/10277 3 cycles)*

Although attitudinal changes were commonly reported, some mappers raised concerns about the length, intensity and location of the training, and how well it prepared them to implement DCM and lead change in practice. For example, some participants perceived DCM implementation to not be covered to a sufficient level during the training, leaving them feeling poorly equipped to implement it back in their workplace.

*“The main thing that I think the training lacks at the minute is implementation, because it’s sort of done on the last day, there’s a few ideas, but not a huge amount.” (50013/10277 1 cycle)*

Some mappers also reported feeling worried or overwhelmed by the volume and pace of the material covered, as well as the expectations to undertake ‘homework’ in the evening after a full day of training.

*“You didn’t have an inch to lose any concentration span because you knew you were sitting a test at the end of each session … it was intense, but it was good.” (50167/10483 1 cycle).*

*“We would sit three or four hours on a night just trying to take it all in … like homework if you like, to try and catch up with myself and do the homework that they set us as well.” (50010/10095 1 cycle).*

Keeping pace with the content was particularly difficult and tiring for non-native English speakers.

*“For me, because English is not my native language … It was quite difficult to understand because the material was very fast.” (58747/10446 3 cycles).*

A number of mappers reported that the training did not cover all of the skills they felt they needed to implement DCM with computer and IT, report writing and practice development skills particularly described as being required, but not taught on DCM training.

“*… you’re given this training, you are quite motivated, you do, you are given a lot of tools to help with dementia, but you’re told get on with it. And the success or failure of that is down to yourself as a manager... and your support from your organisation.” (50018/10269 3 cycles).*

#### Expectations versus reality of the role

Most mappers were initially motivated to undertake DCM training and implement it in practice. Some were aware of the pressure and challenges that implementing DCM might cause and were realistic about what the process might be like.

*“It always struck me as something that wasn’t supposed to be easy. You know, it was supposed to improve the residents’ lives and improve the way staff work and that sort of thing can’t be easy.” (50013/10107 1 cycle).*

However, despite the information provided at the time of recruitment, several mappers reported that they had not fully understood the DCM process or the role and had entered into it with unrealistic expectations. This impacted on their motivation and confidence to lead DCM when they came to become involved in DCM in practice.

*“We kind of got misled, I’ll be honest … a course came up and it was like if you want to learn more about dementia, which I did … and before we knew it we had to go this university and do these massive tests.” (50,010/10096 1 cycle).*

Mappers often referred to the complexity and intensity of the DCM process, which was particularly challenging in the context of the added time pressures that resulted from being part of an RCT.

*“I ended up being off ill by the time I’d finished because I was just so shattered … I had just got so run down.” (58930/40002 3 cycles).*

In some cases, mappers originally recruited using the extended process and criteria set up for the RCT, were unable to attend training and alternative mappers were sometimes hastily selected by managers, at short notice before training. Despite completing the same application form and consent process, these mappers frequently did not understand the role as fully as those recruited earlier and were often less skilled in terms of the mapper criteria. It is thus unsurprising these mappers reported feeling particularly unprepared and sometimes unmotivated for the role, after the trial.

*“They picked two other people to go, and then about three days before they were due to go they backed out. So me and [name] got slapped onto it really, not really wanted to do it...” (10666/10300 0 cycles).*

Two mappers were recruited in each home and expected to work on DCM together throughout the trial. However, in reality, when one of the mappers left the care home, the remaining mapper frequently reported struggling or feeling disengaged with the process, which became far more challenging and daunting to complete alone.

*“The second mapper just resigned and she was gone. So I was doing everything on my own, and it was quite difficult to be honest.” (50024/10349 1 cycle).*

Thus, there was variability in how prepared and motivated mappers reported feeling for the role and to lead practice change through DCM.

### Transferring knowledge into practice

Mappers discussed how able they felt to transfer their knowledge of the intervention into implantation in practice under three sub-themes: *Developing and maintaining skills*; *Motivation through seeing benefit* and *Engaging and transferring learning to the wider staff team.*

#### Developing and maintaining skills

Transferring knowledge learned on the training into applying DCM in practice was challenging for most mappers. Transfer often took time and practice, which there was limited opportunity for within the confines of a clinical trial and the available time and resources within care homes.

*“It was quite hectic to start with, so we started with a couple of residents and we gradually developed our skills to do it properly.” (50021/10315 1 cycle).*

Mappers also felt that their skills were not maintained in the gap between mapping cycles. The support of an expert mapper to implement cycle one, an additional element provided by the trial, was a crucial component of the learning process for many. However, completing cycle two without expert mapper support and after a gap of 3-months since cycle 1 was particularly challenging as evidenced by the large numbers of homes (52%) who did not manage more than this first cycle.

*“It was such a long break from having [expert] in that if you’re not using it you lose it.” (50028/10394 1 cycle).*

Not all aspects of the DCM process were viewed as equally easy to implement. For many mappers the observational component was felt to be relatively straightforward and enjoyable to implement while the other components of the process, in particular data analysis and report writing were seen as extremely challenging. Mappers frequently reported difficulties with computer use and general writing skills and confidence; abilities mappers are expected to possess and that were detailed in the trial selection criteria, but which many of those trained did not have to the required degree to be able to successfully implement DCM.

*“I enjoyed doing the mapping, thoroughly enjoyed all of that bit. I just didn’t like the rest of it.” (50019/10181 1 cycle).*

*“The report writing was horrific to be honest.” (50069/10476 2 cycles).*

*“I couldn’t have done it without [second mapper] because she’s computer literature. We worked it out but it took us a lot longer …*” *(50167/10484 1 cycle).*

#### Motivation through seeing benefit

Mappers who had undertaken some DCM activity, overwhelmingly reported enjoying conducting the observations of practice and predominantly described the observations as providing a positive opportunity for them to gain insight and empathy into life for the residents they cared for. This helped them to see the value DCM could offer to improve practice, for example by observing approaches that worked or practices they wanted to change and provided motivation to persevere even when struggling with implementation.

*“I have to say, that first map I was bored silly and that made me think we are not doing anywhere near enough for these residents. Yes, we’re ticking all the boxes in terms of care, they’re well looked after, you know, everything is up to date in terms for that person. But what are we doing here to keep their well-being on a good level?” (50069/10475 2 cycles).*

*“We both sat there and I could feel myself boiling at one point … you could see their [the residents’] faces looking, and we were both sat there going why don’t you just look over and say hello when you walk through?” (50031/10456 2 cycles).*

However, other mappers gave ambiguous examples of how they felt practice had been changed, which may provide an explanation for the variable motivation mappers demonstrated to continue with mapping during and post-trial.

*“We mapped it and then afterwards we put this, put it in the care plan, so now we have introduced a person-centred care.” (50011/10160 1 cycle).*

#### Engaging and transferring learning to the wider staff team

For DCM to be successful it required engagement and support of the whole staff team. This was more straightforward in some homes than others. For some care homes, engaging staff was difficult at the start, as staff teams were unfamiliar with DCM or were sceptical of the process and despite what mappers’ felt were their best efforts, some staff did not appear to understand what DCM was, or perceived it to lack benefits.

*“I found the first briefing session for the first map really good. The staff were all really engaged, and [mapper 2] and I had just finished the training, so we were like really positive about it.” (50069/10475 2 cycles).*

*“Even though we had information up and we’d spoke to people, I think that the staff didn’t really know what was going on.” (50028/10394 1 cycle).*

*“Some of the comments we’ve had are that it’s pointless, it’s a waste of time.” (50010/10095 1 cycle).*

Turning around negative staff perceptions was, however, felt to be possible with deployment of appropriate leadership and good communication skills.

*“If you are in a care home and you ask staff what the main problem is, they will always say there’s not enough staff, we need more staff to help with our job, and that came up every single feedback session. You have to chair it quite carefully so you can move that away to something constructive, because there isn’t endless pots of money to suddenly employ a load more people.” (50,018/10277 3 cycles).*

In contrast in other settings, initially positive staff engagement decreased over time, often impacting on whether subsequent DCM cycles were completed.

*“The second time, we held a meeting and nobody came.” (50010/10096 1 cycle).*

For some managers who were also mappers, DCM brought a realisation of how much work they needed to do with their staff team to be able to implement DCM and the practice changes they wanted.

*“You realise it’s not that you have to rebuild the building, you have to completely change your staff.” (50018/10277 3 cycles).*

Overall DCM appeared to provide greater benefits for the mappers as individuals than for development of the wider staff team. Mappers discussed difficulties with transferring knowledge and understanding they had gained through DCM training and implementation to colleagues. When asked if they felt that DCM had impacted on staff practice or behaviour change, a few mappers were able to provide specific ways this had happened, the majority said yes but were unable to provide examples of how, and a number said that they felt the benefits were personal to them rather than transferable to staff.

“*Not much other than maybe what I’ve passed on … I think it would be beneficial for them to be taught what we were … but other than that I’d say no not really” (50069/10476 2 cycles).*

*“Yeah I mean I think a few of the staff became a bit more patient” (50013/10107 1 cycle).*

*“[DCM] helped me, you know, more than my staff.” (50024/10349 1 cycle).*

*“I’m hoping it’s increased their understanding, if nothing else, of what they [residents] need. Whether or not it’s increased their confidence I’m not sure” (58930/40002 3 cycles).*

Therefore, developing and maintaining skills was found to be difficult for many staff in the context of the complexity of DCM and the resources available to support its use. Ability to engage staff in the process was largely determined by mappers’ abilities to utilise a range of leadership skills. It was unclear for most mappers if and how DCM changed other staff member’s knowledge or practice, with benefits often felt to be personal to the mappers.

### Sustaining DCM

Sustaining DCM was discussed in relation to three sub-themes: *Resources and support; Conflict/fit with usual role* and *Fit with home’s future plans.*

#### Resources and support

Resources and support for mapping were a common concern for mappers and impacted sustainability of DCM and practice change implementation. A lack of support within the home to fulfil their responsibilities was problematic. Covering shifts due to staff sickness or being taken ‘off the floor’ were consistently mentioned as barriers to implementing DCM, although in homes with good support this was less of a barrier. In many cases managers were viewed as not caring about DCM when difficulties in time allocation were experienced.

*“She [the manager] just weren’t interested, we kept saying to her ‘you need to cover our shifts so we can do this’.” (50010/10095 1 cycle).*

*“Time consuming, but good … we’re quite fortunate that because we’re only small but we’ve got quite supportive staff, we just made sure we had extra on so that we didn’t have to be taken off too much.” (50069/10476 2 cycles).*

In some cases, lack of support meant that mappers completed some or all aspects of DCM in their own time, usually on their days off. This relied on the goodwill of the mappers and was not an expectation of the trial.

*“I couldn’t do it in my normal working hours. I had to do my normal working hours plus a lot of extra hours because we were short. I was sometimes doing 40 odd hours a week, then coming in and trying to do the typing up on top of that.” (58930/40002 3 cycles).*

There were high levels of staff and manager turnover meaning changes during the implementation period. This led to difficulties for mappers, particularly when support from previous managers and staff did not continue.

*“… losing the staff who actually were a great encouragement to do it … we had the new staff coming in and it wasn’t easy to reach them, take time [sic].” (50021/10315 1 cycle).*

The impact of resource issues was not limited to mappers’ ability to implement the DCM process, but also the subsequent action plans. These could be interrupted when staffing levels were low, workloads high or there were emergencies to be attended to.

*“We needed to have someone to spend time with her to help her to read because of the eye difficulties, and she was very ill and very sleepy most of the time. We had an extra carer which we could allocate to spend the time with her, but when we were short of staff … we couldn’t manage.” (58747/10446 3 cycles).*

#### Conflict/fit with usual role

Mappers who were also managers generally benefitted from having more control over the rotas within the care home, meaning they were more easily able to plan cycles into their workload. However, they also experienced difficulties allocating time without being called away for other responsibilities, despite delegating these. These participants also reported boundary issues, having to judge when to include issues highlighted during observations in general DCM feedback and when to see these as capability issues to be dealt with from a managerial perspective.

*“It’s my home, it’s my responsibility and normally If that was a normal day and I heard that, you know, I wouldn’t have it, they’d be in the office immediately with door shut going ‘now, we don’t do that with residents, we don’t speak to them in that way’ … but I couldn’t do it that day until I had the feedback session, I had to generally say it, you know, which I found difficult.” (50069/10475 2 cycles).*

Those with senior roles such as nurses, or team leaders/senior care workers, were often the person who staff came to for support, or were the only staff member who could undertake certain tasks (e.g. administering medication) which was difficult to negotiate whilst mapping, as they were often expected to leave the DCM role to assist.

*“You can’t ignore everything else going on around you, you know people are knocking on the door ‘I know you’re mapping soon I know I’m not supposed to disturb you, but I really need to see you urgently’.” (50167/10483 1 cycle).*

Mappers in roles that had an emphasis on interaction with residents sometimes felt that DCM impacted negatively on those relationships and other mappers felt they lacked the motivation or desire to continue to use DCM.

*“It took me away from being with the residents, and because I’m only here for three days a week, I value that time and they value the time with them as well.” (50167/10484 1 cycle).*

*“I’m very stressed with this computer, I’m not happy to do it anymore … I feel sometimes myself like I’m too old for things, you know like the young they already know these things but for me it’s a kind of struggle … I found myself falling from the moon.” (58747/10447 3 cycles).*

#### Fit with the care home’s future plans

The sustainability of DCM was discussed, with 16 of the mappers, representing twelve of the 15 care homes where at least one DCM cycle had been completed. Despite many having struggled with implementation, twelve mappers said they hoped to use DCM again in the future and only four said they would definitely not use it. A number of mappers had specific ideas of how DCM might fit into the home’s future plans, for example using DCM to develop more personalised care plans for new residents or troubleshooting, (i.e. when a resident experienced behavioural changes). Some mappers discussed ideas for adapting DCM to make it fit their organisation’s needs, such as doing shorter ‘mini’ maps on several days in a week.

*“If people are unsure what we can do for an individual, if there might be initial problems or maybe somebody’s not settling in, or if there’s a change of behaviour …” (50028/10394 1 cycle).*

Given the burdens mapping placed on mappers, one reported challenging their manager over continued use of DCM, as they felt action plans already developed had not yet been implemented.

*“She [Manager] said the other day ‘we’re going to carry on doing this mapping’ and I said ‘well we need to know that something’s going to come out of it’ … it’s alright mapping and saying this needs to be done, but something needs to be done doesn’t it?” (50,010/10096 1 cycle).*

Of the four mappers who did not plan to use DCM going forward, reasons related to lack of support for it within the home’s future plans, and perceived lack of need, as they could identify areas for practice development without engaging in the full mapping cycle.

*“I think once you’re a mapper you don’t really need to stand there and fill in all the tick boxes and everything all the time. I think your observations are much more broader and you’re more aware of things going on.” (50167/10483 1 cycle).*

Therefore, sustained use of DCM relied on mappers feeling there was a positive fit between DCM and their role within the care home and with the care home’s future plans.

## Discussion

While previous studies have explored staff perceptions of implementing complex interventions in care homes, few have focussed on the unique perspective of those staff tasked with implementation leadership, and none have examined this in relation to DCM implementation specifically. The present study identified that for trained mappers, the process of implementing DCM centred around three key areas: Preparedness to lead; Transferring knowledge into Practice; and Sustaining DCM.

In discussing implementation of interventions in health and care services, Damschroder and colleagues [28] developed the Consolidated Framework For Intervention Research (CFIR) in which they identify five major domains. One domain concerns the individual characteristics of those implementing the intervention, which is comprised of five constructs: An individual’s knowledge and beliefs about the intervention; self-efficacy; individual stage of change; individual identification with organisation; and other personal attributes. These have specific relevance when considering the findings of this study.

*Preparedness to lead* related to the initial impressions of the role and DCM training that care home staff received and aligns with the CFIR construct of ‘knowledge and beliefs about the intervention’, which includes the values individuals placed on the intervention and their enthusiasm for its use. The majority of those recruited reported volunteering or readily agreeing to become mappers. Overwhelmingly participants reported DCM training to be interactive, engaging, informative and a positive and enjoyable experience, even if at times fast-paced and intensive. Interactive group-based training that encourages discussion and supports reflection has been identified as an important component of successful psychosocial interventions [10] and DCM training met these criteria. Initial learner reactions to training can increase receptivity to change, learning, and motivation [29, 30] and thus also likelihood of implementation of learning in practice. Therefore, due to positive training experiences, initially most mappers were enthusiastic and willing to undertake leadership of DCM.

However, for a considerable number of mappers their initial perception of the mapper role differed to the reality once they were required to *Transfer knowledge into practice*. This theme aligns to the CFIR constructs of ‘self-efficacy’ and ‘personal attributes’. Despite their initial positive reactions to training, many mappers reported that once they began implementation, they did not feel confident or adequately prepared for what was required. Provision of support from an external expert during cycle one helped with some consolidation of learning (see [26]). However, participants recommended that DCM training should cover components such as report writing, feedback and action planning more comprehensively. However, achieving a balance between provision of more comprehensive training, to support care home staff to feel better prepared to implement complex interventions involving practice change, against practical issues such as costs and release of staff to attend longer training, is likely to be challenging. Considering the CFIR constructs of self-efficacy and personal attributes, our study indicated that many of the staff recruited as mappers did not have the wealth of personal attributes (organisational skills, literacy, numeracy, confidence, authority etc) necessary to lead practice change in care home settings. This combined with the complex nature of DCM meant that for many mappers feelings of self-efficacy were low. Thus, mappers did not have the requisite resources to implement DCM effectively.

Due to DCM’s complexity, mappers’ discussed the need to be well-practiced through starting to using DCM immediately post-training and the need for continued honing of skills between mapping cycles. This is a core component identified as being crucial to sustainable intervention implementation in other research [31] and has resonance with the CFIR construct of ‘individual stage of change’. Individual stage of change refers to the phase an individual is in as they progress towards skilled and sustained use of the intervention. This study indicated that mappers felt a need to quickly and continually use DCM in order to retain skills and increase competence and confidence in its use. However, achieving this within the resources required versus available, was challenging. For most, it was difficult to identify ways that gaining mastery in use of a complex intervention like DCM, within a resource constrained environment, could be achieved. This suggests it may be pertinent to consider ways that DCM might be simplified, for example by reducing the complexity of the coding, removing the need to use computers for data analysis and reporting and teaching mappers about different approaches to implementation that may be more achievable within care home settings (e.g. short maps with verbal feedback only).

Motivation to keep trying to *Transfer knowledge into practice* was gained through mappers perceiving practical benefits from implementing the observational component. However, for some these were outweighed by challenges with the IT and paperwork aspects of data analysis, feedback and action planning, as well as difficulties engaging the wider staff team. A unique finding from our study, likely to also be transferable to other complex interventions, is that despite DCM pertaining to be a whole home intervention, training two care home staff to use the method did not appear to provide the requisite conditions for this learning and thus for the effects of the intervention to be cascaded across the care home. Our study indicates the wider staff team need to have a more extensive knowledge of person-centred care, and greater understanding of DCM, than it seems feasible for most newly qualified mappers to provide. To successfully implement psychosocial interventions active engagement of the whole staff team is required [32], which may be achieved through including all staff in training and intervention implementation to promoting learning and supporting sustainability [10]. Likewise a study of characteristics of care homes associated with highly person-centred care highlighted the importance of a philosophy of care shared across the staff team, wide staff support for person-centred care and broad staff access to dementia training [33]. It seems therefore, that widespread, care home-level support for delivery of person-centred care should be a prerequisite for, rather than an outcome of DCM. Mappers in this study indicated it would be beneficial for all staff to undergo DCM training, given the benefits they felt it provided for increasing empathy and understanding of person-centred care. However, given the pragmatic and cost considerations, this is unlikely to be feasible. One solution then, may be to provide a wider programme of training as part of implementing DCM within an organisation. This might include a shorter version of the DCM course for staff who will participate in implementation of practice change, and more comprehensive development for those tasked with leadership and application of the DCM tool and process.

Another key factor was *DCM sustainability* and within this, how well this fitted with available care home resources, its culture and future plans, as well as the fit with the mappers ongoing role in the care home. This aligns with the CFIR domain of ‘individual identification with organisation’, which refers to how individuals perceive their organisation and their degree of organisational citizenship or commitment. Much of this hinged on the support from management for DCM in terms of ensuring ongoing provision of adequate resources and fit with both the mappers’ ongoing role and the care home’s future plans. The barriers presented by resource constraints, and the importance of managerial support for PCC to underpin intervention implementation, has been identified as crucial across a range of reviews and studies considering implementation of complex interventions [10, 21, 34–36]. Thus, provision of more detailed information to care home providers about the required setting conditions, culture, ethos and future plans needed to underpin intervention implementation, may assist them to identify if a particular care home is one in which DCM is likely to be successful.

### Strengths and limitations

While this is the first study to explore the experiences of care home staff trained to use DCM in any depth, and on such a scale, there are several limitations. The study presents data from participants from 18 of the 31 intervention care homes in the clinical trial and therefore may not represent the views of those homes who were not included in the process evaluation. It also only includes the experiences of the mappers who were still employed by the care home after final data collection and thus does not contain the experiences of those who left the role during the trial. The interviews asked mappers to reflect retrospectively on their experiences across the 16-months of the trial and so this may have affected the ways in which mappers perceived the implementation issues. Interviewing mappers after each DCM cycle, for example, may have provided a more thorough and different perspective.

### Recommendations for practice

There are several implications for practice arising from the study. Firstly, before embarking on DCM care homes must undertake preparatory work including all staff being trained in person-centred care and committed to developing person-centred practice, a clear implementation plan and a commitment across management to ensure adequate resources are dedicated to the process of sustainable DCM implementation. Ongoing support from someone with expertise in DCM use is also important for the majority of mappers upon training completion. Selection of the right staff to train as mappers is also essential. This must include consideration of the wider range of personal skills and qualities needed to lead practice change, alongside willingness to undertake the role. Thus, attendance at DCM training should reflect the end point of a longer process of planning and preparation for its use. Without these things in place DCM will be difficult to implement sustainably.

## Conclusions

The evidence from this study indicates that only a small number of care homes, which already have the requisite setting conditions, are likely to be able to successfully and sustainably implement DCM. These are likely to be the homes with a pool of staff with the skills and personal attributes to lead practice change using DCM, supportive leadership and management and a stable and engaged staff team who have an in-depth knowledge of and a commitment to deliver person-centred care. These are conditions unlikely to be present in many care homes, particularly those who might benefit most from an intervention such as DCM to help them to improve care. This may also to apply to many other similar complex interventions. The study suggests that adaptations to the DCM tool, process and training should be considered to reduce the complexity and burden of implementing the tool and to better prepare care homes, staff and mappers to undertake practice development using the method. Alternatively, different models of DCM implementation, which are not reliant on care home staff to deliver this alone should be considered.

## Data Availability

The datasets generated and/or analysed during the current study are not publicly available due to consent given but are available from the corresponding author on reasonable request.

## Abbreviations

CFIR: Consolidated Framework For Intervention Research
DCM: Dementia Care Mapping
